# Heat Shock Proteins and Antioxidant Genes Involved in Heat Combined with Drought Stress Responses in Perennial Rye Grass

**DOI:** 10.3390/life12091426

**Published:** 2022-09-13

**Authors:** Md Atikur Rahman, Jae Hoon Woo, Yowook Song, Sang-Hoon Lee, Md Mahadi Hasan, Md Abul Kalam Azad, Ki-Won Lee

**Affiliations:** 1 Grassland and Forage Division, National Institute of Animal Science, Rural Development Administration, Cheonan 31000, Korea; 2 State Key Laboratory of Grassland Agro-Ecosystems, College of Ecology, Lanzhou University, Lanzhou 730000, China; 3 Department of Biochemistry and Biotechnology, University of Science and Technology Chittagong, Khulshi 4202, Bangladesh; 4 ABEx Bio-Research Center, East Azampur, Dhaka 1230, Bangladesh

**Keywords:** climate change, heat shock protein, gene, antioxidant, heat, drought, interactome, rye grass

## Abstract

The frequent occurrence of heat and drought stress can severely reduce agricultural production of field crops. In comparison to a single stress, the combination of both heat (H) and drought (D) further reduce plant growth, survival and yield. This study aimed to explore the transcriptional responses of heat shock protein (HSP) and antioxidant genes under H combined D stress in perennial rye grass (PRG). The results demonstrated that oxidative stress indicators (hydrogen peroxide, lipid peroxidation) significantly increased, particularly in the case of combined H and D treatment, suggesting that oxidative stress-induced damage occurred in plants under the combined stresses. Transcriptional responses of heat shock protein 70 (*HSP70*), heat shock protein 90-6 (*HSP90-6*), and the mitochondrial small heat shock protein HSP26.2 (*HSP26.2*) occurred rapidly, and showed high level of expression particularly under H and D stress. Antioxidant genes including ascorbate peroxidase (APX), glutathione reductase (*GR*), monodehydroascorbate reductase (*MDHAR*), dehydroascorbate reductase (*DHAR*), catalase (*CAT*), copper–zinc superoxide dismutase (*Cu/ZnSOD*), peroxidase (*POD*), ferredoxin–thioredoxin (*FTR*), thioredoxin (*Trx*), 2-cysteine peroxiredoxin (*2-Cys Prx*) showed response to combined H and D, followed by either D or H stress alone in rye grass. An interactome map revealed the close partnership of these heat shock protein genes and antioxidant genes, respectively. These candidate genes were predominantly linked to stress responses and antioxidant defense in plants. These findings may advance our understanding about the HSP and the antioxidant genes underlying combined abiotic stress response and tolerance in perennial rye grass.

## 1. Introduction

The environmental temperature is continuously rising due to global climate change, which has an adverse effect on crop growth, phenology and productivity [1]. Global crop production and food security are seriously threatened by heat and drought stress [2]. The co-occurrence of heat and drought is very common in tropical and sub-tropical regions causing significant economic losses in agriculture [3]. Drought and temperature stress affect a substantial area of agricultural fields, having a significant impact on crop growth and productivity [4]. Dryland areas are exhibiting widely due to increasing temperatures, so this problem is predicted to further worsen globally due to climate extremes and unsustainable management of resources, soil and crop genotypes [5]. Therefore, uncovering the physiological and molecular mechanisms of the combined stressess is crucial to creating suitable strategies for sustainable crop production. 

Plant exposure to high temperatures and/or drought generates excess free radicals (O_2_^•−^, H_2_O_2,_ OH), cellular injury, and oxidative stress, which inhibit vital cellular and metabolic processes [6]. These combined effects retard plant growth, lead to wilting and even death in most plant species. Therefore, exploration of the molecular mechanisms associated with plant response to heat and/or drought stress can be useful for improving plant stress tolerance through molecular breeding. There are genetic and genomics studies suggesting that many proteins and antioxidant genes are involved in heat and drought stress response in plants [7,8]. These candidate proteins and genes protect plants from abiotic stresses via cellular homeostasis, regulation of metabolic proteins, signaling and antioxidant defense [9,10,11]. The different abiotic stresses such as heat, cold, drought and flood can induce several common cellular disorders including membrane injury, oxidative damage and protein denaturation. During stress stimulation, signaling molecules regulate the downstream effectors, primarily protein kinases (PPK) and transcription factors (TF), which lead to alterations in gene expression and protein/enzyme activities, thereby boosting plant defense systems. Accordingly, plants contain restored unsaturated fatty acids, ROS-scavenging antioxidants, molecular chaperone and compatible solutes, which protect plants from the adverse effects of abiotic stresses [12]. Several studies are available on gene expression induced by short-term (0 to 24 H) heat or drought stress [4,13]. However, present studies on prolonged drought and heat in grass species will be valuable in addition to the previous studies. Plants have evolved tolerance mechanisms to cope with these adverse effects by changing expression of stress-responsive genes with antioxidative function [14]. 

Heat shock protein (HSP) and antioxidant genes are the key regulators of stress responses in plants. There are several members of the heat shock proteins (HSPs) family, including the *HSP60* (chaperonin), *HSP70, HSP90*, *HSP90-6, HSP100*, and small HSP (*sHSP*) [9]. The heat shock protein genes (*Hsp17.8*, *smHsp23, Hsp26* and *Hsp101*) were reported to induce their expression under several abiotic stresses including drought, heat, salinity, and cold [7,8,15,16]. However, members of the HSP family play a vital role in protecting plants from stress through re-establishing normal protein structure and cellular homeostasis [9]. Plants bear very efficient antioxidant enzyme mechanisms that detoxify abiotic stress-induced reactive oxygen species (ROS) [17,18]. Plants activate a group of antioxidant enzymes including superoxide dismutase (*SOD*), catalase (*CAT*), ascorbate peroxidase (*APX*), glutathione reductase (*GR*), monodehydroascorbate reductase *(MDHAR)*, dehydroascorbate reductase (*DHAR)*, glutathione peroxidase (*GP*), peroxidase (*POD*). Glutathione peroxidase (*GPX*) and *APX* are two important ROS-scavenging enzymes that protect plants from hydrogen peroxide (H_2_O_2_)-derived cellular damage by catalyzing the reduction of H_2_O_2_ [19,20]. The substrate specificity and catalytic mechanisms of peroxidases are more diverse. The APX enzyme catalyzes the H_2_O_2_-dependent oxidation of L-ascorbate, and its properties place it between class I (e.g., cytochrome c peroxidase) and class III (e.g., horseradish peroxidase) peroxidases [21]. However, ferredoxin–thioredoxin (*FTR*), thioredoxin (*TRX*), and peroxiredoxin (*PRX*) have been reported as components of the redox system in plants [22], which regulate redox signaling during plant development and stress adaptation, and mitigate abiotic stress-induced oxidative damage in plants [17,18,19,23]. Several reports suggest that enzymes connected to the ascorbate–glutathione (AsA-GSH) pathway alleviate abiotic stress-induced oxidative damage in plants [19,23,24]. Despite these recent advancements, little attention has been given to the expression patterns of HSPs and the antioxidant genes in forages or grass species in response to combined abiotic stress with prolonged duration. 

Perennial rye grass (*Lollium perenne* L.) is a cool-season forage that is extensively cultivated in warm temperate to sub-tropical regions of the world [25]. Perennial rye grass (PRG) faces high temperatures (>38 °C) in summer and low temperatures (<−10 °C) in winter [26]. PRG represents an important resource of the livestock system, with its high palatability and digestibility attracting interest from the dairy and sheep feed industry. PRG is a rapid-growing grass species with high yield potential and is widely cultivated in South Korea. Thus, improving the traits associated with high temperature and drought adaptability can improve stress tolerance along with high yield potential in Korean local environments that have not yet been thoroughly investigated in PRG. Therefore, the objectives of this study were to investigate the effect of high temperature combined with drought stress at the molecular level, and exploration of potential responses in the antioxidant defense system of PRG. In this study, the results concerning the physiological and molecular responses of PRG under heat and drought stress greatly enhanced our understanding about PRG adaptation to the combined stresses of high temperature and drought. 

## 2. Materials and Methods

### 2.1. Plant Cultivation and Treatment

Seeds of perennial rye grass (*Lollium perenne* L. cv Bison) were placed into plastic trays for germination, and maintained growth for 1 week, after which the seedlings were transferred to soil (3:1; field soil: potting mixed) containing pots. The growth chamber conditions were maintained at 25 °C under white fluorescent light (480 μmol m^−2^ s^−1^) with a 14-h photoperiod and 60–65% relative humidity. Plants were grown for 2 weeks and then the water supply was suspended to induce drought stress. The reduction in pot soil moisture was measured every day during drought treatment and was recorded as percentage of field capacity (FC). Mild drought stress was initiated in PRG at day 13 with soil moisture content in pots measuring 23.95% FC. At this point, one more set plant was prepared for heat treatment at 37 °C for 72 h. The treatments were as follows; control (C), heat 37 °C combined with drought (H + D), heat 37 °C (H), and drought (D). Each treatment was considered with five independent biological replications.

### 2.2. Determination of Hydrogen Peroxide Accumulation

Hydrogen peroxide (H_2_O_2_) was determined according to the protocol used previously [27]. Briefly, 100 mg plant tissue was homogenized with 50 mmol potassium phosphate buffer (pH 6.8) containing 1 mmol hydroxylamine (a catalase inhibitor). The homogenate was centrifuged at 12,000 rpm for 20 min, and 0.6 mL supernatant was placed in a new tube, with subsequent addition of 0.6 mL of 20% H_2_SO_4_ containing 0.1% (*v*/*v*) titanium chloride. The mixture was centrifuged at 12,000 rpm for 15 min. The supernatant was taken and the optical density (OD) was measured at 410 nm using a UV-spectrometer (Spectra MAX i3X, San Jose, CA, USA). The H_2_O_2_ accumulation was calculated considering an extinction co-efficient of 0.28 µmol^−1^ cm^−1^.

### 2.3. Measurement of Malondialdehyde Content

Malondialdehyde (MDA) content was measured using thiobarbituric acid (TBA) following the method described by Ref [28]. Shortly, 100 mg of plant tissue was mixed with 20% (*w*/*v*) trichloroacetic acid (TCA). The mixture was vortexed well then centrifuged for 15 min at 13,000 rpm. In a new tube, 0.5 mL supernatant was added to 0.5 mL TCA containing TBA 0.5% (*v*/*v*), and 100 μL butylated hydroxytoluene (BHT) 0.4%(*v*/*v*). The tube was incubated at 95 °C water bath or heating block for 30 min, and then quickly cooled in ice for 5 min. The homogenate was centrifuged for 15 min at 13,000 rpm, and the sample OD was checked at 532 nm and 600 nm, respectively, using the UV-spectrometer (SpectraMAXi3X, San Jose, CA, USA). Finally, the absorbance was recorded at 600 nm, and non-specific absorbance was subtracted. Finally, the MDA content was calculated by its extinction coefficient of 155 mM^−1^ cm^−1^.

### 2.4. RNA Extraction and Real-Time PCR Analysis

Total RNA was extracted from leaf tissue using RNA extraction kit (QIAGEN, Germantown, MD, USA). Briefly, 100 mg plant tissue was homogenized with extraction buffer containing 1% β-mercaptoethanol (β-ME). After subsequent washing steps, total RNA yield was recovered. RNA concentration was checked via UV/Vis spectrophotometer (UV Drop-99, Taipei, Taiwan), and RNA concentration ≥ 300 ng/μL was considered for subsequent molecular analysis. RNA quality was assessed using agarose gel electrophoresis. The first stand of cDNA was synthesized from RNA using cDNA synthesis kit (Bio-Rad, Hercules, CA, USA). Real-time PCR analysis was performed via CFX-96™ real-time PCR (BIORAD) for the expression of heat shock protein and antioxidant genes using gene specific primers (Table 1). The PCR protocol was set to amplify the target genes as follows: 95 °C for 30 s, followed by 40 cycles at 95 °C for 5 s, 60 °C for 30 s, and extension at 60 °C for 1 min. Housekeeping gene *Actin* was considered as internal control. Gene expression was analyzed following the 2^−∆∆Ct^ method [29]. 

### 2.5. Interactome and Gene Expression Partners Analysis

The interactome and co-expression partners of heat shock protein and antioxidant genes were generated using STRING server (https://string-db.org/) and visualized via Cytoscape [30]. The analyses were also checked with model plant Arabidopsis homolog to annotate local network cluster, functional partners, molecular function, protein domain, and gene co-expression. 

### 2.6. Statistical Analysis

The physiological and gene expression data were statistically analyzed using analysis of variance (ANOVA), and the Tukey test was selected to compare the treatment means. Probability level (*p* ≤ 0.05) was used to determine whether a difference was statistically significant. In addition, the GraphPad Prism program (version 9.0) was used to construct the graphical figures. All the results were presented as mean value ± standard error (S.E.) of at least three biological replications.

## 3. Results

### 3.1. The Effect of Heat and Drought Stress on Plant Morphology and Membrane Oxidation

**Figure 1:**
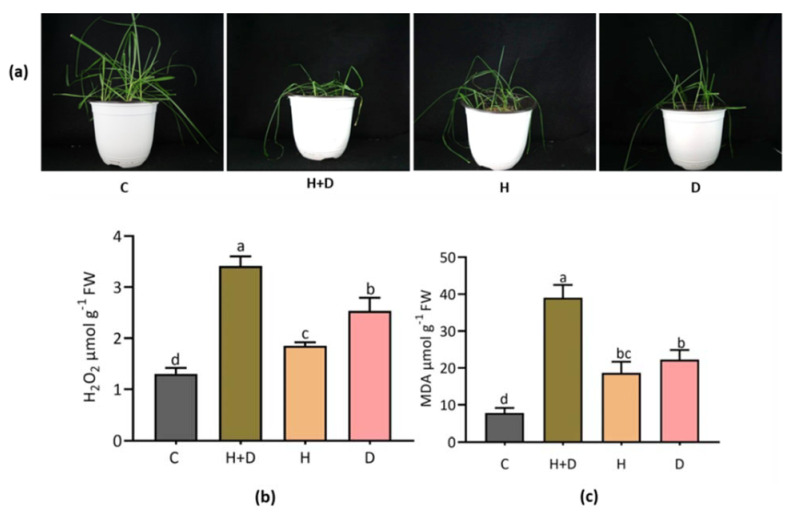
Heat- and drought-induced stress severity and regulation of oxidative stress indices in perennial rye grass. Morphological changes of plants following combined (H + D) and single stress treatment (**a**); accumulation of hydrogen peroxide (H_2_O_2_) (**b**); and malondialdehyde (MDA) (**c**) in response to different stresses. Letters C, H, and D represent control, heat and drought, respectively. Different letters above the column bar indicate significant difference among the means ± SD of treatments (n = 3) at a *p* < 0.05 significance level.

Perennial rye grass (PRG) exhibited morphological differences in response to heat (H) and drought (D) stress. The H combined with D stress, showed severe plant wilting compared to H and/or D alone and control plants (Figure 1a). Hydrogen peroxide (H_2_O_2_) is an indicator of stress-induced free radical formation, and malondialdehyde (MDA) is extensively used for determining oxidative stress-induced lipid peroxidation in cells. The H combined with D stress exhibited a high level of H_2_O_2_ compared to H or D stress alone (Figure 1b). A significant level of MDA was observed in H combined with D stress treatment while no significant difference was found between the H or D stress alone treatments (Figure 1c). 

### 3.2. Expression of Key Heat Shock Protein Genes Involved in Cellular Homeostasis 

**Figure 2:**
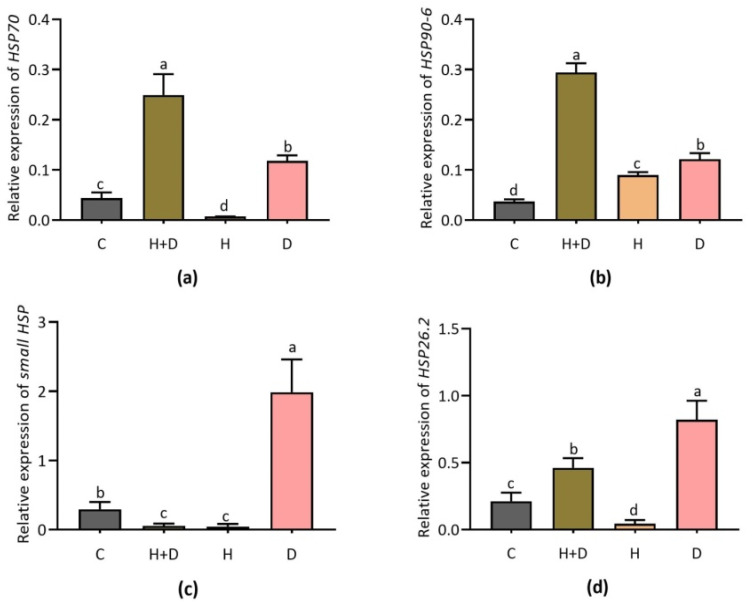
Expression of *HSP70, HSP90-6, small HSP*, and *HSP26.2* in response to combined (H + D) and single stress treatment. Expression of *HSP70* (**a**); *HSP90-6* (**b**); *small HSP* (**c**); and *HSP26.2* (**d**). Different letters above the column bar indicate significant difference among the means ± SD of treatments (n = 3) at *p* < 0.05 significance level.

As shown in Figure 2, H and D significantly induced the transcripts of several key heat shock protein genes. Heat shock protein 70 (*HSP70*) transcripts were highly induced in response to combined H and D compared to control and single stress treatment (Figure 2a). *HSP90-6* showed a similar response with it being highly expressed in the combined treatment while lightly induced under H or D stress alone, respectively (Figure 2b). Chloroplastic small heat shock protein (*sHSP*) was induced significantly in response to single D stress, while no significant expression was observed under combined (H + D) and single H stress (Figure 2c). Single D exhibited a significant expression of mitochondrial small heat shock protein *26.2* (s*HSP 26.2*) compared to H + D and single H stress (Figure 2d).

### 3.3. Expression of Ascorbate-Cycle-Dependent Genes

**Figure 3:**
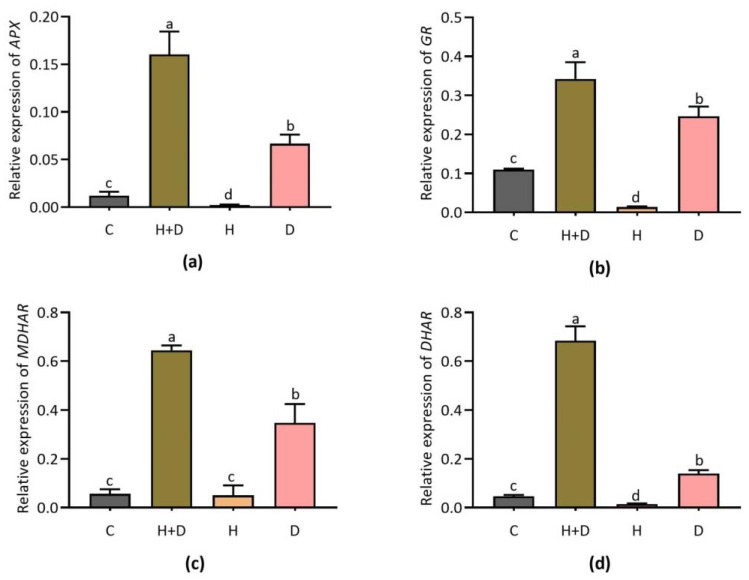
Expression of ascorbate-cycle-dependent genes *APX, GR, MDHAR*, and *DHAR* in response to combined (H + D) and single stress treatment. Expression of *APX* (**a**); *GR* (**b**); *MDHAR* (**c**); and *DHAR* (**d**). Different letters above the column bars indicate significant difference among the means ± SD of treatments (n = 3) at *p* <0.05 significance level.

The expression patterns of ascorbate-cycle-dependent genes were found to be induced mostly by combined stress (H + D) and single D stress (Figure 3). Combined stress greatly induced *APX* transcripts compared to control and single stress (Figure 3a). Glutathione reductase (*GR*) showed a similar expression pattern while exhibiting no considerable expression in response to H stress (Figure 3b). Ascorbic acid (AsA)-mediated reduction/oxidation (redox) regulation related key gene *MDHAR* was highly induced under combined stress compared to the single D stress condition (Figure 3c). A similar expression pattern was exhibited by *DHAR* under combined stress while it was lightly induced by single D stress (Figure 3d).

### 3.4. Effect of Heat and Drought Stress on Expression Patterns of Glutathione (GSH)-Cycle-Dependent Genes

**Figure 4:**
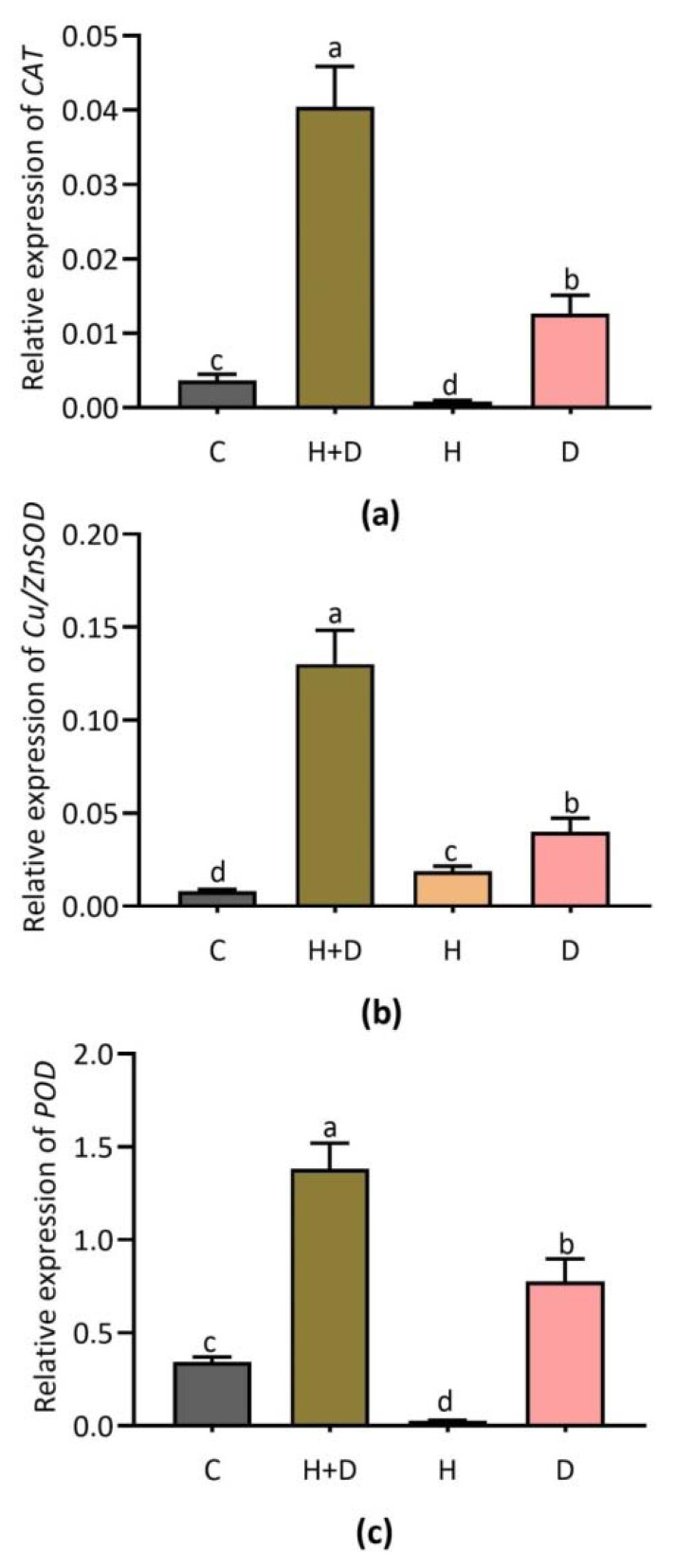
Expression of glutathione (GSH)-cycle-dependent genes *CAT, Cu/ZnSOD*, and *POD* in response to combined (H + D) and single stress treatment. Expression of *CAT* (**a**); *Cu/ZnSOD* (**b**); and *POD* (**c**). Different letters above the column bars indicate significant difference among the means ± SD of treatments (n = 3) at *p* < 0.05 significance level.

The combined heat and drought and single D stress conditions mediated the expression of GSH-cycle-dependent genes (Figure 4). Key gene *CAT* was highly expressed by combined stress compared to control while it was lightly induced by single D stress (Figure 4a). The expression patterns were significantly different under the combined stress condition in comparison to H and D stress alone, respectively (Figure 4b). Heat and drought significantly induced *POD* transcripts compared to control while no considerable response under single H stress was identified (Figure 4c).

### 3.5. Effect of Heat and Drought Stress on Key Genes Involved in Antioxidant Defense 

**Figure 5:**
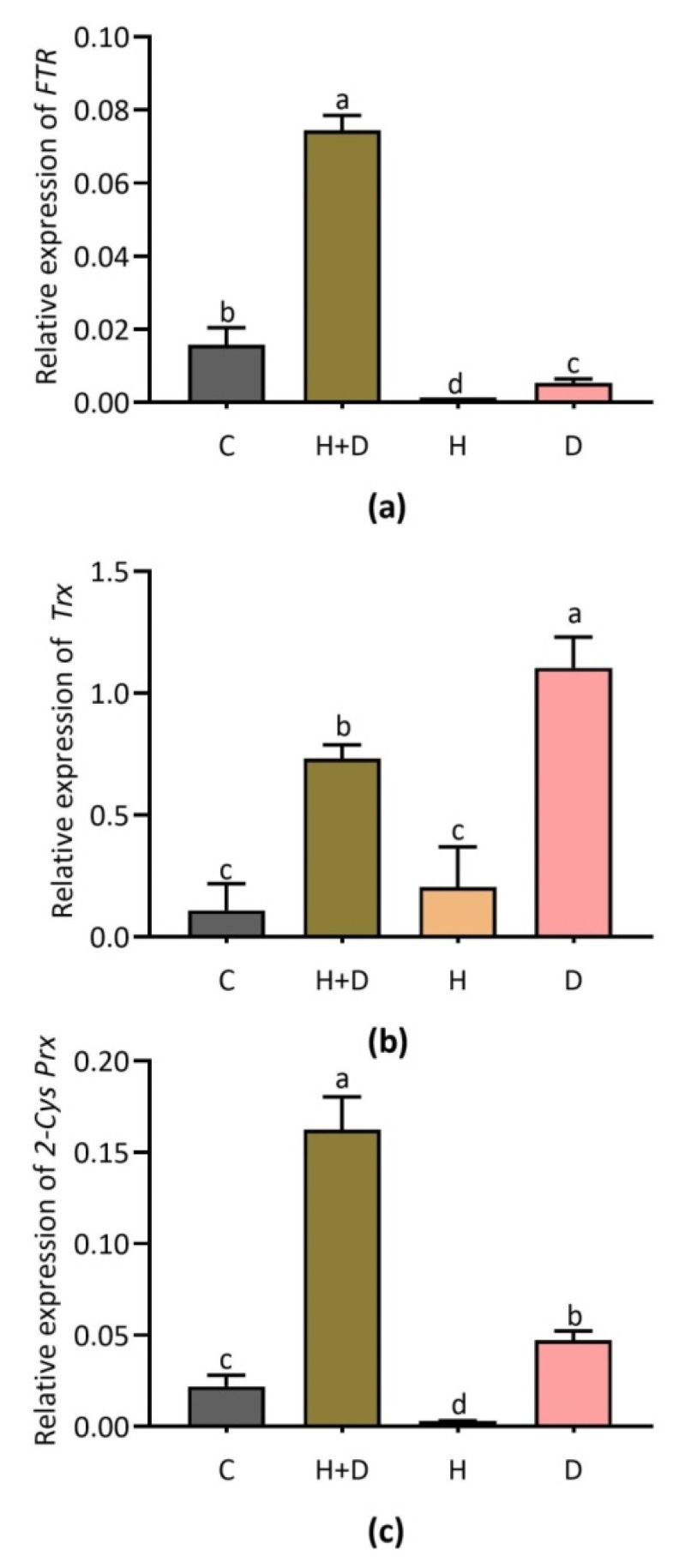
Expression of antioxidant defense genes *FTR*, *Trx*, and *2-Cys Prx* in response to combined (H + D) and single stress treatment. Expression of *FTR* (**a**); *Trx* (**b**); and *2-Cys Prx* (**c**). Different letters above the column bars indicate significant difference among the means ± SD of treatments (n = 3) at *p* <0.05 significance level.

Mostly combined (H + D) stress, and single D stress induced the expression pattern of gene transcripts *FTR, Trx* and *2-Cys Prx* (Figure 5). The expression of *FTR* was highly induced by heat combined with drought stress relative to control plants while being induced lightly under single D stress (Figure 5a). *Trx* was highly expressed in response to single D stress, followed by combined stress, and small induction of this gene was found under single H stress (Figure 5b). The *2-Cys Prx* gene displayed significant expression under combined stress, followed by single D stress while no considerable response was found under single H stress (Figure 5c).

### 3.6. Analysis of Interactome Map of Heat Shock Protein and Antioxidant Candidates 

**Figure 6:**
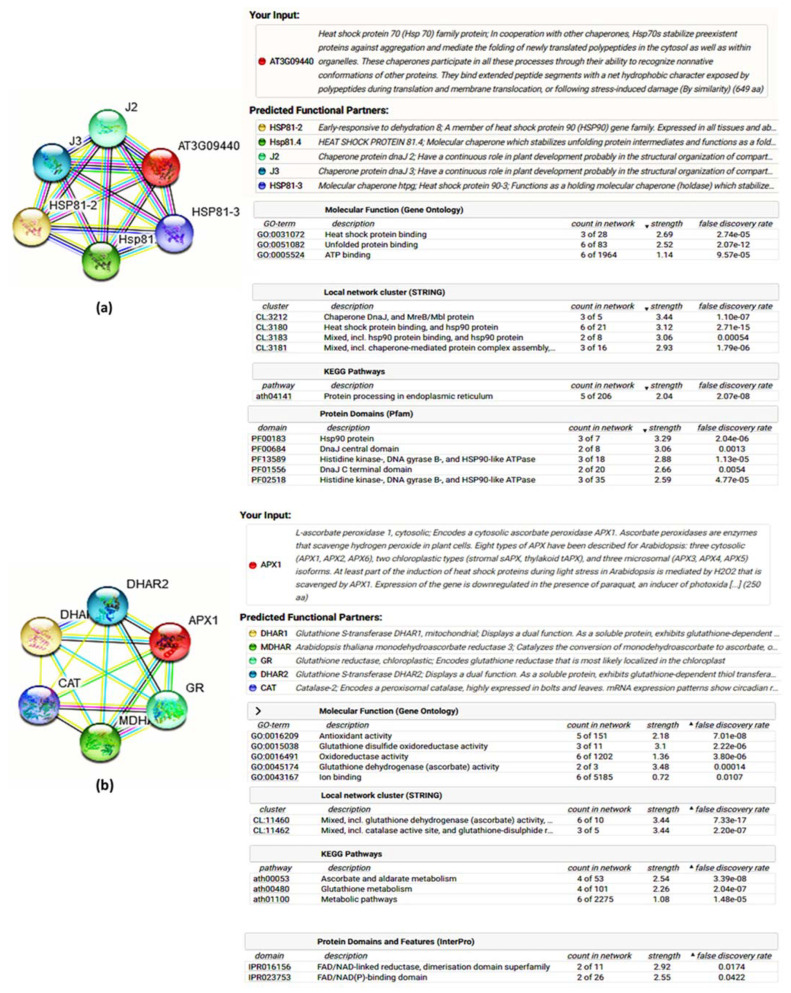
Interactome map analysis of heat shock proteins and antioxidant candidates. Interactome map with interaction partners of *HSP70* (**a**); and *APX1* (**b**) genes with *Arabidopsis thaliana* network.

The best closely related interaction partners of HSP70 and APX were selected from the interactome analysis. The *HSP70* protein was found to have a functional relationship with *HPS 81-2* (Heat shock protein 81-2), *HPS 81.4* (Heat shock protein 81.4), *J2* (chaperone protein dnaj2), *J3* (chaperone protein dnaj 3) and *HPS 81-3* (Heat shock protein 81-3) (Figure 6a). *APX1* showed partnership with *DHAR1* (dehydroascorbate reductase 1), *MDHAR* (monodehydroascorbate reductase), *GR* (glutathione reductase), *DHAR2* (dehydroascorbate reductase 2), and *CAT* (catalase) (Figure 6b).

### 3.7. Gene Co-Expression Analysis of Heat Shock Protein and Antioxidant Candidates

**Figure 7:**
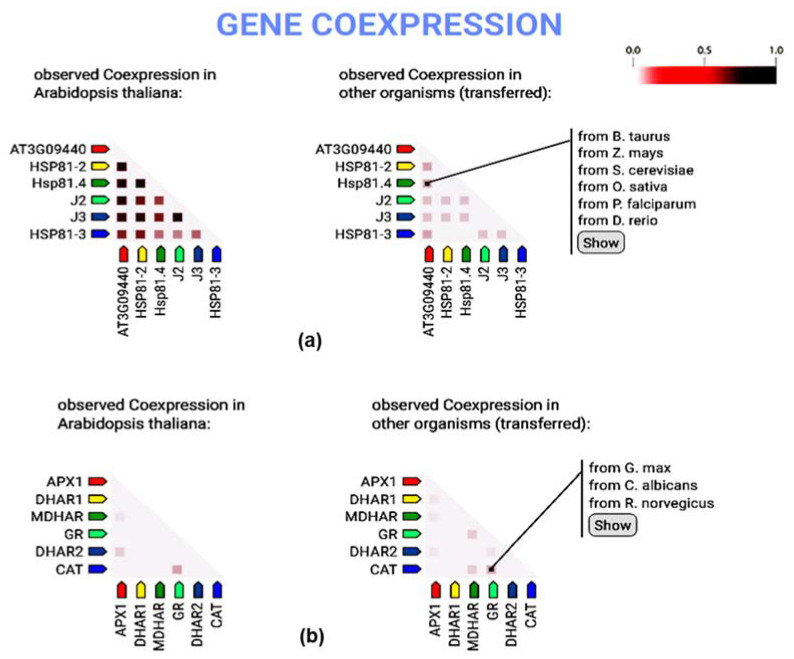
Gene co-expression analysis of heat shock protein and antioxidant candidates. Co-expression of heat shock protein (**a**); and antioxidant candidates (**b**) with *Arabidopsis thaliana*. In the triangle-matrices above, the color intensity indicates the level of confidence that two candidates are functionally associated, given the overall expression data in the organism.

The gene co-expression analysis revealed a correlation among the heat shock proteins and antioxidant candidate genes from *Arabidopsis thaliana* and genes from perennial rye grass. *HSP70, HPS81-2* (Heat shock protein 81-2), *HPS81.4* (Heat shock protein 81.4), *J2* (chaperone protein dnaj2), *J3* (chaperone protein dnaj3) and *HPS81-3* (Heat shock protein 81-3) showed a strong positive correlation with each other (Figure 7a). *APX1* exhibited a positive correlation with *DHAR2* (dehydroascorbate reductase 2), and light correlation with *MDHAR* (monodehydroascorbate reductase), while *GR* (glutathione reductase) showed positive correlation with *CAT* (catalase) (Figure 7b).

## 4. Discussion

### 4.1. Heat Combined with Drought Stress Severely Induced Wilting and Accumulation of Oxidative Stress Indices in Perennial Rye Grass

Combined stress (H + D) severely inhibited normal physiological indices and led to the wilting of PRG seedlings. Apart from the oxidative stress indicator, the elevation of H_2_O_2_ and MDA suggests that the oxidative stress and cellular injury occurred in response to combined and/or single stress treatment in PRG. The increase in H_2_O_2_ and MDA levels coincided with plant damage. Accumulation of MDA under stress conditions usually reflects damage of cell membrane in plants. Oxidative stress and physiological damage occurred under prolonged heat (36 °C) in PRG [31]. In our study, we found similar physiological responses in combined stress as well as single H and D stress conditions in PRG. Previous studies on heat and drought tolerance mechanisms in PRG revealed that they were dependent on the series of physiological and molecular processes, as well as stress sensitivity of PRG cultivars [32,33].

### 4.2. Heat Shock Protein Genes Responded to Heat and Drought Stress in Perennial Rye Grass

Heat tolerance depends on genetic variation among the rye grass cultivars, and activation of heat shock proteins (HSPs) under heat stress [34]. Apart from the heat stress, HSPs are known to be expressed during several abiotic stresses including heat, cold, drought, hypoxia, and UV-light [35]. Several heat shock transcription factors including *HSFA, HSFB*, and *HSFC* played key role in plant abiotic stress tolerance *HSFC1b* as a positive regulator of heat stress in PRG [33]. Relevant study for heat shock factors (*Hsfs*) were reported in tall fescue and perennial rye grass leaves using RNA-Seq analysis and mentioned *Hsfs* are important regulators of stress-response in plants [26]. We found transcriptional response of heat shock protein 70 (*HSP70*), heat shock protein 90-6 (*HSP90-6*) induced in response to the combined H and D stress, while mitochondrial smHSP and heat shock protein HSP26.2 (*HSP26.2*) induced particularly in D stress. These findings suggest differential expression patterns of HSPs/molecular chaperones in different abiotic stresses. However, diverse responses of HSP are not surprising in plants. Previous study suggests that smHSP showed diverse response to abiotic stress in higher plants [12]. Under stress conditions, smHSPs were induced and prevented the aggregation of non-native proteins, which were involved in cell survival under stress [9,12] However, comparing our findings to those that have already been published suggests this information might be useful for future gene function and molecular breeding research for improving stress tolerance in grass and related crop species.

### 4.3. ROS-Scavenging Related Antioxidant Genes Involved in Heat and Drought Stress Responses in Perennial Rye Grass

Reactive oxygen species (ROS) can either damage or activate defense responses in organisms. Competition related to stress-induced ROS generation and response of the scavenging system ultimately facilitate adaptability in an organism. Among other locations, ROS generation occurs in the mitochondria of cells [36]. An intimate relationship between antioxidant genes and abiotic stress responses was identified in grass species [37]. In the present study, we found that alterations in ROS-scavenging antioxidant genes (*CAT, Cu/ZnSOD*, and *APX*) expressions were strongly induced in response to H and D stress in PRG. The transcriptional regulation of *SOD, CAT* and *APX* was linked to cellular ROS-scavenging activity against heat- and drought-induced damage in plants. Our study results were supported by the previous study on *CAT2, SOD* and *APX* expression in barley, wherein the *SOD* and *APX* genes were upregulated and involved in controlling severe drought stress [38]. In addition, the expressions of *SOD* and *POD, APX* and *CAT* were altered in response to heat stress, which helped to mitigate damage associated with heat and low Cd^2+^ combined stress via antioxidant defense regulation in rice [39]. Furthermore, *SOD* was documented as an integral candidate and considered a front-liner antioxidant against O_2_^•−^ produced as a secondary product of ETC [40]. In our current study, the expressions of *Cu/ZnSOD, POD, APX* and *CAT* were found to be induced under conditions of combined and/or single H and D stress. These findings together suggest that *Cu/ZnSOD, POD, APX* and *CAT* are key candidates in antioxidant defense, which might be useful in molecular breeding strategies for developing stress-tolerant PRG.

### 4.4. Interactome Discoveries and Background of Functional Genomics Studies in Grass, and Related Plant Species 

The interactome networks of a specific gene provide biological information about its associations, which is useful for screening the close partnership with functional analysis using several platforms. The STRING platform’s interactome map and gene co-expression analyses revealed that previous gene partners associated with PGR-heat shock protein and antioxidant genes, respectively. The interactome map presented the top five most predicted partners of *HSP70*, and antioxidant gene *APX.* These interactome discoveries might provide an important platform for functional genomic studies in grass and other related crop species.

**Figure 8:**
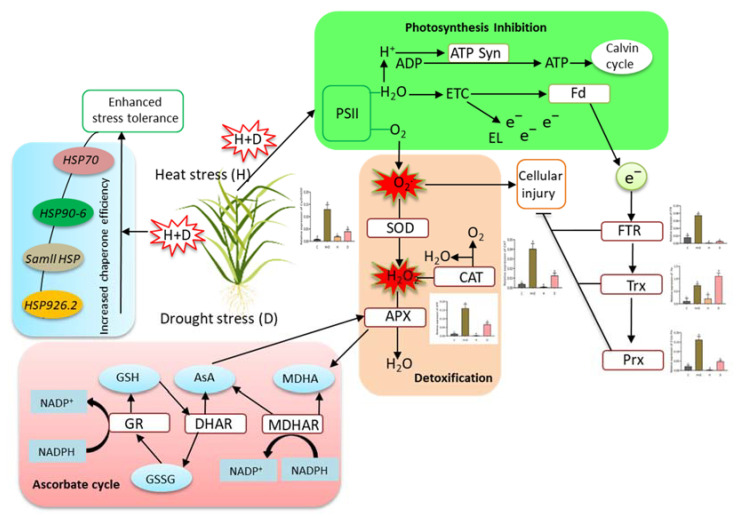
Mechanistic insights of heat shock protein and antioxidant candidate genes involved in heat combined drought stress responses in perennial rye grass. Abbreviations: H^+^, hydrogen molecule; ADT, adenosine diphosphate; ATP, adenosine triphosphate; H_2_O, water; ETC, electron transport chain; e-, electron; EL, electrolyte leakage; Fd, ferredoxin; PSII, photosystem II; O_2_, oxygen molecule; O_2_^•−^, superoxide radical; SOD, superoxide dismutase; CAT, catalase; APX, ascorbate peroxidase; FTR, ferredoxin–thioredoxin reductase; Trx, thioredoxin; Prx, peroxiredoxin; MDHA, monodehydroascorbate; AsA, ascorbate; GSH, glutathione; MDHAR, monodehydroascorbate reductase; DHAR, dehydroascorbate reductase; GR, glutathione reductase; NADP^+^, oxidized form of the electron donor nicotinamide adenine dinucleotide phosphate; NADPH, nicotinamide adenine dinucleotide phosphate; HSP70, heat shock protein 70; HSP90-6, heat shock protein 90-6, HSP; heat shock protein; HSP26.2, heat shock protein 26.2.

In summary, we presented the mechanistic insights of heat shock proteins and antioxidant genes involved in heat combined drought stress responses in perennial rye grass (Figure 8). Heat and/or drought stress restricted normal physiological processes in PRG. As a consequence, oxidative stress indicators (H_2_O_2_, MDA) were elevated following heat and/or drought stress, which suggest oxidative stress-induced damage in plants. In contrast, a group of heat shock proteins and antioxidant genes showed higher expressions under combined (H + D) stress or single D stress. However, H or D stress significantly regulated the expression of *SOD, CAT* and *APX* genes, which were involved in the detoxification process. This process shares a link with the ascorbate cycle (Figure 8). On the other hand, heat shock protein genes responded dramatically to combined stress, and/or single D or H stress treatment (Figure 8). The interactome map revealed a close partnership between these heat shock protein genes and antioxidant genes, which indicates a strong link between specific genes and the function of their predicted partners. These incorporated findings have greatly enhanced our insight regarding the HSP and antioxidant genes involved in abiotic stress response and tolerance in perennial rye grass.

## Figures and Tables

**Table 1:** A list of *Lolium* primers used for the gene expression analysis.

Gene Name	Forward	Reverse	Accession Number
*HSP70*	CACGATTGGCCCATTCCAAC	CGTCTCGTGGTGCATCATCT	JF747479
*HSP90-6*	AAGCTCGGTTGCATGGAAGA	GGAGCATTTTTGGCACTGCT	XM_047191399
*small HSP*	GGTGAAGATGCGGTTCGACA	ATGTCGTAGGAGCTGACGC	XM_047229918
*HSP26.2*	GGCGAGAAGGATGCATGGAA	CTCGATGGCGATCTGGAACA	XM_047189720
*APX*	GACGTGATTCCTCGGTTTGC	TCAGAGGATCACGGGTCCAT	JF747449
*GR*	TGGCTTTGGGTGGACTTACG	ACAGCTTGCCATCCACACTA	XM_047233600
*MDHAR*	CGGTTCAGATGCTGCAAACA	CCATGCAGTGTTTTTCCGGG	JF747451
*DHAR*	ACCAACGATGGAACAGAGCA	AGTGAATCTGGGACAGACCA	JF747452
*CAT*	ACAAGTTCGACTTCGACCCG	CAGCATCTTGTCGTCGGAGT	JF747381
*Cu/ZnSOD*	CGGTGACACAACTAACGGGT	CCCAACAACTGCATTTGGGC	XP_047061758
*POD*	CGGTTCCATTCGAGGCATGA	TCTTGCTTGCGGTGGTAGAG	XM_047215214
*FTR*	CCGTCGTCATCAAGGGACTT	GAAATCGTTGTCGGGGGTGA	XM_047224812
*Trx*	AACTCCTCTCGTGTCAGAGC	TGGGGCGTTACCTTCCAAAA	EL664102
*2-Cys Prx*	GGAGTGGACGATTTGCCGTT	ACTCATCGTGTCTGTCGCTG	XM_047225703

## Data Availability

Not applicable.
